# Emerging multi-port soft tissue robotic systems: a systematic review of clinical outcomes

**DOI:** 10.1007/s11701-024-01887-w

**Published:** 2024-03-30

**Authors:** Yit J. Leang, Joseph C. H. Kong, Zahin Mosharaf, Chrys S. Hensman, Paul R. Burton, Wendy A. Brown

**Affiliations:** 1 Oesophago-Gastric and Bariatric Surgical Unit, Department of General Surgery, The Alfred Hospital, Melbourne, VIC Australia; 2 Department of Surgery, Central Clinical School, Monash University, 55 Commercial Road, Melbourne, VIC 3004 Australia; 3 Colorectal Unit, Department of General Surgery, The Alfred Hospital, Melbourne, VIC Australia

**Keywords:** Robotic surgery, Colorectal, Urology, Cholecystectomy, Surgical outcomes, Systematic review

## Abstract

Multiple novel multi-port robotic surgical systems have been introduced into clinical practice. This systematic review aims to evaluate the clinical outcomes of these novel robotic systems to conventional laparoscopic technique and established da Vinci robotic surgical platforms. A literature search of Embase, Medline, Pubmed, Cochrane library, and Google Scholar was performed according to the PRISMA guidelines from 2012 to May 2023. Studies comparing clinical outcomes of novel multi-port robotic surgical systems with laparoscopic or the da Vinci platforms were included. Case series with no comparison groups were excluded. Descriptive statistics were used to report patient and outcome data. A systematic narrative review was provided for each outcome. Twelve studies comprised of 1142 patients were included. A total of 6 novel multi-port robotic systems: Micro Hand S, Senhance, Revo-i MSR-5000, KangDuo, Versius, and Hugo^™^ RAS were compared against the laparoscopic or the da Vinci robotic platforms. Clinical outcomes of these novel robotic platforms were comparable to the established da Vinci platforms. When compared against conventional laparoscopic approaches, the robotic platforms demonstrated lower volume of blood loss, shorter length of stay but longer operative time. This systematic review highlighted the safe implementation and efficacy of 6 new robotic systems. The clinical outcomes achieved by these new robotic systems are comparable to the established da Vinci robotic system in simple to moderate case complexities. There is emerging evidence that these new robotic systems provide a viable alternative to currently available robotic platforms.

## Introduction

The global adoption of robotic surgery continues to rise in different surgical specialties like colorectal [1–3], urology [4, 5], bariatrics [6–8], upper gastrointestinal [9–11] and gynecology [12–14]. While a large proportion of the robotic systems currently installed are the well-established da Vinci robotic surgical system (Intuitive Surgical Inc, California, USA), various manufacturers have developed and introduced alternative robotic systems. A few examples are the Revo-i (Meerecompany, Inc., Seongnam, Republic of Korea) [15, 16], Senhance (formerly ALF-X) (Asensus Surgical, North Carolina, USA) [17, 18], Versius (CMR Surgical, Cambridge, UK) [19], Micro Hand S (Wego, Qingdao, China) [20], Hugo^™^ RAS (Medtronic, MN, USA) [21, 22], and Hinotori^™^ surgical robot system (Medicaroid Inc., Kobe, Japan) [23, 24].

Most of these newer robotic surgical systems have been developed with distinctive capabilities such as haptic feedback, modular system, single port operating and implementation of artificial intelligence. In addition, there is also a target for a value-driven healthcare by reducing the device acquisition and ongoing operational cost. These are promising developments especially for ‘robot-naïve’ healthcare systems contemplating to adopt robotic surgical technologies.

Over the last few years, most publications on these novel robotic systems were early model development, preclinical results, feasibility studies and small case series. However, some centers have started publishing their results comparing these novel platforms to conventional laparoscopic approaches and even the da Vinci surgical system.

The aim of this study is to systematically review the existing literature on the clinical outcomes of these newer robotic surgical systems.

## Methods

This systematic review of literature and meta-analysis was conducted in accordance to the recommendations of the Preferred Reporting Items for Systematic Reviews and Meta-analyses (PRISMA) guidelines [25]. No ethical approval was required. This systematic review was registered in The International Prospective Register of Systematic Reviews (PROSPERO) with the registration number CRD42023475626.

### Electronic search

An electronic search was performed on the following databases: Embase, Medline, Pubmed, Cochrane library and Google Scholar independently by two reviewers on 1st May 2023. The search period was set from year 2012 to May 2023 to identify all published and indexed studies comparing clinical outcomes of newly developed multi-port soft tissue robotic surgical systems against laparoscopic (lap) or da Vinci (DV) robotic approach. A combination of “MeSH” and non- “MeSH” search terms: robotic surgery, robotic console, robotic surgical system, robotic surgical device, laparoscopic surgery, and laparoscopic procedure were used. A manual search of the reference lists of relevant studies was performed to identify additional studies.

### Study selection

Two reviewers (Y.L., Z.M.) screened the studies independently to identify articles for potential inclusion. Studies were screened by their titles, abstracts, followed by their full texts. Any conflicts were resolved by consensus.

Studies were considered eligible for inclusion if patients were adult (18 $\ge$ years old) undergoing robot-assisted soft tissue surgery using newly developed robotic surgical systems with clinical outcomes being compared against laparoscopic or da Vinci robotic approach. Only articles published in English were considered. Studies with insufficient outcome reporting, duplicated data, missing either a laparoscopic arm or da Vinci arm as comparison, case series or reports were excluded.

### Data extraction

The primary outcomes of interest were clinical outcomes, including but not limited to the following: surgical complication rate: Clavien–Dindo grading (CD), length of stay (LOS), estimated blood loss (EBL), conversion rate being defined as conversion from the intended robotic approach to any other approaches or a different robotic platform, and standard oncological outcomes in cancer resection studies.

Data were extracted from studies that met the eligibility criteria. Parameters extracted included title, first author, year of publication, country where the study was conducted, study design, number of patients, patient characteristics, type of surgery and outcomes of interest.

### Risk of bias assessment

The quality of the included studies was assessed by two reviewers independently using the Newcastle–Ottawa scale (NOS) for non-randomized studies [26] or Jadad scale for randomized control trial (RCT) [27].

### Data analysis

Descriptive statistics were used to report patient and outcome data. A meta-analysis was not performed due to the heterogeneity of the procedures and reported clinical outcomes. A systematic narrative review was provided for each outcome.

## Results

### Literature search

**Fig. 1:**
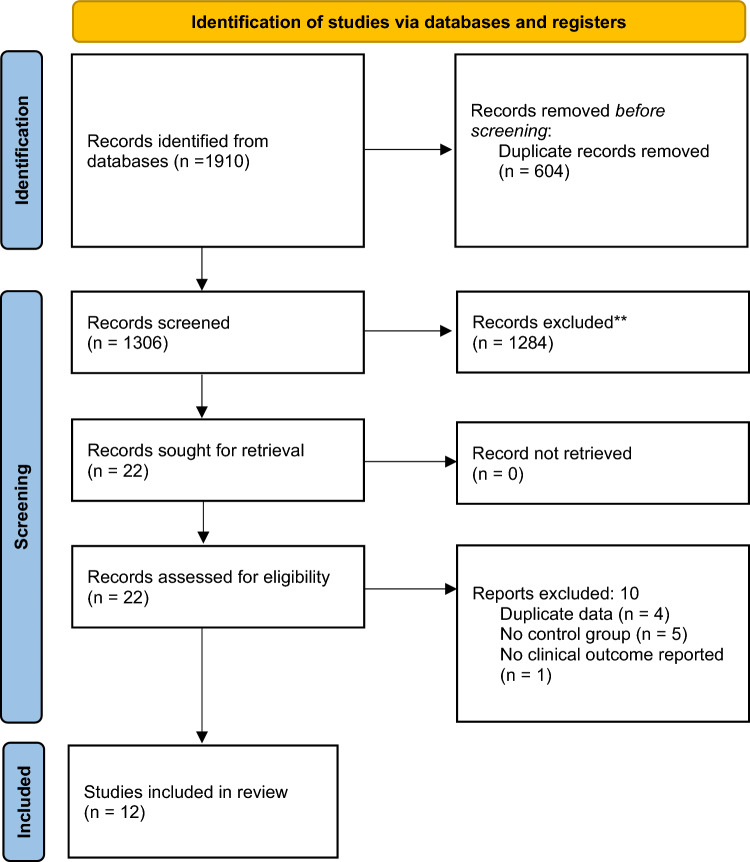
PRISMA 2020 flow diagram of literature search

The literature search conducted on the electronic databases revealed 1904 records. Six additional studies were identified from bibliography citation (Fig. 1). After excluding duplicates and records that did not match the main topic, 22 articles were evaluated in full text. Ten articles were excluded as 4 contained overlap data from studies published earlier, 5 articles had no control group, and 1 article did not report clinical outcomes. Twelve studies were included for qualitative analysis [28–39].

### Characteristics and quality of included studies

A total of 1142 patients were presented in the 12 studies with a range between 22 and 168 patients in each study (Table 1). All the studies were published within the last 5 years with 7 studies reported from China and 5 other studies from Croatia, South Korea, Pakistan, India, and Lithuania, respectively. There were 6 newly developed robotic systems being compared in the 12 studies including: Micro Hand S robotic system (Wego, Qingdao, China), Senhance surgical system (Asensus Surgical, NC, United States), Revo-i, model: MSR-5000 (Meerecompany, Inc., Seongnam, Republic of Korea), KangDuo surgical robot (Suzhou KangDuo Robot Co., Ltd., Suzhou, China), Versius robotic surgical system (CMR Surgical, Cambridge, UK), and Hugo^™^ RAS system (Medtronic, Minneapolis, MN, USA).

**Table 1:** Characteristics of eligible studies

Study ID	Country	Time period	Design	Surgical system comparison		Patients		Procedure type	Quality of article (NOS)
				Control	Intervention	Control	Intervention		
Colorectal									
Luo [28]	China	2017–2019	Retrospective	Da Vinci (platform version not specified)	Micro Hand S robotic system	24	21	Sigmoid colon cancer resection (radical)	7
Zeng [29]	China	2018–2019	Retrospective	Laparoscopic	Micro Hand S robotic system	12	10	Right hemicolectomy	7
Wang [30]	China	2015–2018	Retrospective	Laparoscopic	Micro Hand S robotic system	65	40	Total mesorectal excision	7
Liu [35]	China	2017–2020	Prospective	Da Vinci Si/laparoscopic	Micro Hand S robotic system	47/45	43	Total mesorectal excision	6
Urology									
Kulis [34]	Croatia	2019–2020	Prospective	Laparoscopic	Senhance robotic system	61	107	Radical prostatectomy	8
Alip [31]	South Korea	2016–2020	Prospective	Da Vinci Si	Revo-I MSR-5000	33	33	Radical prostatectomy	7
Fan [32]	China	2019–2021	Prospective	Da Vinci Si	KangDuo surgical robot	16	16	Pyeloplasty	7
Hussein [33]	Pakistan	2017–2021	Retrospective	Da Vinci Si	Versius robotic surgical system	114	114	Urology*	7
Li [38]	China	2020–2021	RCT (multi-center)	Da Vinci Si	KangDuo surgical robot	50	49	Partial nephrectomy	J3
Ragavan [39]	India	2021–2022	Prospective	Da Vinci (platform version not specified)	Hugo^™^ RAS	17	17	Radical prostatectomy	8
Hepatobiliary									
Samalavicius [36]	Lithuania	2018–2019	Retrospective (propensity score matched)	Laparoscopic	Senhance robotic system	20	20	Cholecystectomy	8
Wang [37]	China	2019–2020	RCT (multi-center)	Da Vinci Si	Micro Hand S robotic system	84	84	Cholecystectomy	J5

*Urology cases comprised of pyeloplasty, radical nephrectomy, simple nephrectomy, nephrolithiasis, and partial nephrectomy

There were 2 multi-center RCTs, 5 prospective studies, and 5 retrospective studies. All the non-randomized studies were single-center studies. Wang et al. [30] and Liu et al. [35] both report outcomes of total mesorectal excisions (TME) from the same center with overlap of study period from January 2017 to November 2018 (23 months). However, Liu et al.’s study was a prospective trial with different inclusion criteria. Hence, all patients were assumed to be independent and included. Hussein et al. reported outcomes of a mixture of urology cases following a propensity matched analysis. Therefore, outcomes for each procedural types were extracted and grouped accordingly for qualitative comparison and analysis in this review [33].

All 10 prospective and retrospective studies scored 6 or more on NOS, and therefore were deemed good quality studies [28–36, 39]. Two RCTs: Wang et al. [37] and Li et al. [38] scored 3 and 5, respectively, on the Jadad scale indicating moderate and good quality.

## Outcomes

### Colorectal resections

Four studies reported clinical outcomes on colorectal resections: 1 sigmoid resection, 1 right hemicolectomy, and 2 total mesorectal excision (TME). The studies originated from China and all surgeries were conducted using their locally developed multi-port robotic system: Micro Hand S (MH), (Table 2).

**Table 2:**
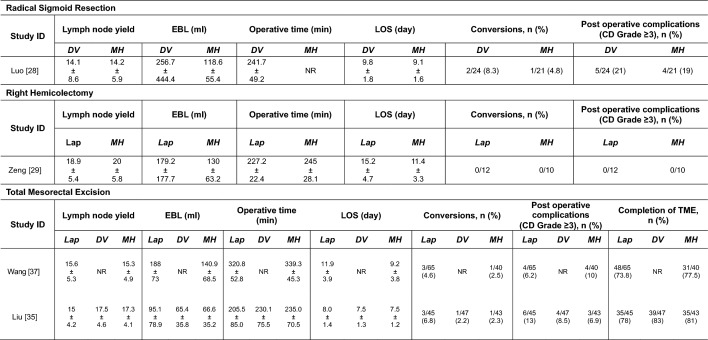
Summary findings in colorectal resections

*EBL* Estimated blood loss; *LOS* length of stay; *CD* Clavien–Dindo; *Lap* laparoscopic; *DV* Da Vinci; *MH* Micro Hand S; *TME* total mesorectal excision; *NR* not reported

In the sigmoid resection study, Luo et al. presented their retrospective cohort study of 45 patients (24 DV versus 21 MH) comparing outcomes of 2 different robotic platforms on radical sigmoid colon cancer resection [28]. All patients had histologically confirmed sigmoid colon carcinoma, no distant metastasis and underwent curative resection by experienced surgeons with a primary colorectal anastomosis. Patients with an American Society of Anaesthesiologists (ASA) classification more than 3 were excluded. The patients’ demographics and tumor characteristics were equally matched. There was no difference in short-term outcomes: lymph node yield, EBL, LOS, conversion rate, and moderate to severe post-operative complications (CD Grade ≥ 3) between the DV and the MH group. Operative time and long-term oncological outcomes were not reported.

Zeng et al. reported their outcomes on laparoscopic versus robotic right hemicolectomy using the MH robotic platform [29]. It was a retrospective cohort of 22 patients (12 lap versus 10 MH). All patients had histologically confirmed adenocarcinoma of the right colon, no distant metastasis and underwent elective curative resection by the same surgical team within the study period with pre-operative bowel preparation. The patients’ demographics and tumor characteristics were well matched. The robotic group had a significantly shorter LOS (11.4 ± 3.3 days vs. 15.2 ± 4.7 days, *p* = 0.046). There was no difference in other short-term outcomes: lymph node yield, EBL, conversion rate and moderate to severe post-operative complications (CD Grade ≥ 3) between the laparoscopic and the MH group.

In terms of rectal resections, Wang et al.’s study [30] retrospectively compared consecutive cases performed by a single surgeon early in the learning curve of both laparoscopic and robotic TME (RTME) using the MH robotic platform. The patients underwent routine investigation for pathological confirmation of rectal carcinoma and staging with both computed tomography (CT) and magnetic resonance imaging (MRI). There were 65 patients in the laparoscopic group versus 40 patients in the MH group. Both groups were evenly matched in general demographics, comorbidity, TNM stage, rate of neoadjuvant chemoradiotherapy (lap 7.7% vs. RTME 12.5%), procedure type (low anterior resection (LAR) and abdominal perineal resection (APR)) and protective ileostomy (lap 51.7% vs. RTME 55%). Clinical outcomes between the 2 groups were not different in terms of completion of TME, lymph node yield, EBL, operative time, LOS, conversions, and post-operative complications (CD Grade ≥ 3).

In contrast, Liu et al. [35] conducted a prospective trial comparing outcomes of 3 different minimally invasive approaches: lap, DV and MH in TME. This was not a randomized trial as patients were free to select their method of surgical resection albeit performed by the same surgeon. The number of patients were similar in all groups. No significant differences were found in general demographics, comorbidity, TNM stage, rate of neoadjuvant chemoradiotherapy and diverting stoma rate. In comparison between the laparoscopic group, DV and MH groups, the laparoscopic group had a higher rate of Hartmann’s (6.7% vs. 0%, 0%, *p* = 0.033) and APR (13.3% vs. 4.3%, 2.3%, *p* = 0.035), higher volume of blood loss [95.1 ± 78.9 ml vs. 65.4 ± 35.8 ml (*p* = 0.037), 66.6 ± 35.2 ml (*p* = 0.041)], lower lymph node yield [15 ± 4.2 vs. 17.5 ± 4.6 (*p* = 0.0310), 17.3 ± 4.1 (*p* = 0.033)], higher rate of conversion to open [6.8% vs. 2.2% (*p* = 0.038), 2.3% (*p* = 0.04)], and higher rate of severe complications, in particular anastomotic leak [13.9% vs. 4.4% (*p* = 0.023), 2.3% (*p* = 0.031)]. Operative time in the lap group was shorter than the robotic groups, DV and MH [205.5 ± 85 min vs. 230.1 ± 75.5 min (*p* = 0.043), 235 ± 70.5 min (*p* = 0.045)].

### Radical prostatectomy

Three studies reported clinical outcomes on radical prostatectomy [31, 34, 39] (Table 3). All 3 studies compared different robotic platforms. Kulis et al. [34] conducted a prospective study comparing the Senhance robotic system (*n* = 107) to laparoscopic group (*n* = 61). Two operating surgeons with limited laparoscopic and robotic radical prostatectomy experience (< 20 cases as primary surgeon) conducted the procedures. The demographics and clinical stage of the prostate tumor were matched. The laparoscopic group was associated with a higher EBL and LOS with no differences in positive surgical margins and post-operative complications (CD Grade ≥ 3). The conversion rate in the Senhance group was significantly higher (8.7% vs. 0%) due to robotic platform issue and anatomical constraints.

**Table 3:** Summary findings in urological procedures

Radical prostatectomy												
Study ID	EBL (ml)		Operative time (min)		LOS (days)		Conversions, *n* (%)		Post operative complications (CD Grade ≥ 3), *n* (%)		Positive surgical margins, *n* (%)	
	Lap	Senhance	Lap	Senhance	Lap	Senhance	Lap	Senhance	Lap	Senhance	Lap	Senhance
Kulis [34]	362.5 ± 272.4 (first 30 cases) 257.5 ± 46.6 (last 31 cases)	287.5 ± 286.2 (first 50 cases) 250 ± 114.1 (last 57 cases)	161.3 ± 83.7 (first 30 cases) 145.8 ± 26 (last 31 cases)	155 ± 70.6 (first 50 cases) 148.2 ± 14.5 (last 57 cases)	6 ± 4.3 (first 30 cases) 4 ± 0.4 (last 31 cases)	4 ± 1.1 (first 50 cases) 3.75 ± 0 (last 57 cases)	0	9/107 (8.4) [2—technical issues with robot, 7—anatomical reasons]	0/61	1/107 (0.9)	14/61 (23)	30/107 (28)

	DV	Revo-i	DV	Revo-i	DV	Revo-i	DV	Revo-i	DV	Revo-i	DV	Revo-i
Alip [31]	206.4 ± 165.9	284.2 ± 262.3	92.4 ± 26.1	126.2 ± 55.2	5.8 ± 2.1	5 ± 1.9	0/33	0/33	1/33 (3)	0/33	15/33 (45)	16/33 (48)

	DV	Hugo^™^	DV	Hugo^™^	DV	Hugo^™^	DV	Hugo^™^	DV	Hugo^™^	DV	Hugo^™^
Ragavan [39]	NR	NR	165 ± 12.1	153.8 ± 24.3	1 ± 0.4	1 ± 0.4	0/17	0/17	0/17	0/17	4/17 (24)	4/17 (24)

Pyeloplasty										
Study ID	EBL (ml)		Operative time (min)		LOS (days)		Conversion, *n* (%)		Post operative complications (CD grade ≥ 3), *n* (%)	
	DV	KD	DV	KD	DV	KD	DV	KD	DV	KD
Fan [32]	10 (5–50)	8 (5–50)	118 ± 31	141 ± 28	4.2 ± 1.6	4.3 ± 1.5	0/16	0/16	2/16 (12.5)	1/16 (6.3)

	DV	Versius	DV	Versius	DV	Versius	DV	Versius	DV	Versius
Hussein [33]	47.5 ± 23.6	62.5 ± 21.9	97.5 ± 15.7	101.5 ± 30.6	1.75 ± 1.2	2 ± 0.4	0/25	0/9	NR	NR

Partial nephrectomy										
Study ID	EBL (ml)		Operative time (min)		LOS (days)		Conversion, *n* (%)		Post operative complications (CD grade ≥ 3), *n* (%)	
	DV	KD	DV	KD	DV	KD	DV	KD	DV	KD
Li [38]	30 (20–50)	50 (10–50)	61.5 ± 19.7	69.6 ± 30.9	4 (4–8)	4 (3–10)	0/50	0/49	3/50 (6)	3/49 (6.1)

	DV	Versius	DV	Versius	DV	Versius	DV	Versius	DV	Versius
Hussein [33]	431.3 ± 423.2	275 ± 166.9	155.8 ± 42.6	122.5 ± 19.1	NR	NR	1/4 (25)	0/6	NR	NR

*EBL* Estimated blood loss; *CD* Clavien–Dindo; *LOS* length of stay; *Lap* Laparoscopic; *DV* Da Vinci; *KD* KangDuo; *NR* not reported

Alip et al. [31] published their outcomes of radical prostatectomy in South Korea comparing 2 robotic platforms: DV versus Revo-i developed in South Korea. All surgeries were performed by an experienced robotic surgeon who had performed more than 1000 robotic prostatectomies using the DV platform. A 1:1 propensity score matching analysis was performed using the following co-variates: age, ASA score, body mass index (BMI), previous abdominal and endoscopic surgery, pre-operative prostate specific antigen (PSA), prostate volume, International Society of Urological Pathology (ISUP) grade group, tumor stage, and need for pelvic lymphadenectomy resulting in 33 patients in each group. The Revo-i group had a significant longer operative time (126.2 ± 55.2 min vs. 92.4 ± 26.1 min, *p* < 0.01) but shorter LOS (5 ± 1.9 days vs. 5.8 ± 2.2 days, *p* = 0.036) compared to the DV group. There was no difference in EBL, surgical complications, and surgical margins.

Ragavan et al. [39] published their series of 17 patients who underwent robotic radical prostatectomy with the new Hugo^™^ RAS system (Hugo) platform. Seventeen matching patients who underwent robotic radical prostatectomy using the DV platform were selected from their database as controls for comparison. Demographics, pre-operative PSA, and tumor stage were evenly matched. A shorter operative time was noted in the Hugo^™^ group (153.8 ± 24.3 min vs. 165 ± 12.1 min). No differences in surgical complications, surgical margins, or conversion to open were detected.

### Pyeloplasty

Two studies published outcomes on pyeloplasty [32, 33] (Table 3). Fan et al. [32] reported their early outcomes of 16 patients operated on a newly developed robotic platform: KangDuo (KD) surgical robot versus 16 patients on the established Dd Vinci robotic platform. The 2 groups were evenly matched in terms of demographics, BMI, comorbidity, pathology, and anatomy. There were no differences in operative time, EBL, LOS, success rate and complication rate between the 2 groups. Similarly, Hussein et al. [33] described their early experience and outcomes with the Versius robotic system in comparison to the more established DV system in their center. Patients in the DV group were matched to the same proceduralist performing the same operation on 2 different platforms. There were 25 patients in the DV group and 9 in the Versius group with slightly better outcomes in the DV group in operative time, EBL and LOS.

### Partial nephrectomy

Two studies published outcomes on partial nephrectomy [33, 38] (Table 3). Li et al. [38] conducted a double-center RCT comparing outcomes of partial nephrectomy in patients with T1aN0M0 renal carcinoma between KD and DV. 50 patients were recruited into each group but 1 patient in the KD group did not undergo the procedure due to an equipment sterilization issue. Both groups were well matched in terms of age, BMI, pathology, and anatomical factors. Although the total operative time was similar between the 2 groups, the robot-docking time and suture time per stitch were significantly longer in the KD group (4.26 ± 1.69 min vs. 3.44 ± 1.26 min, *p* = 0.015), (48 ± 15 s vs. 31 ± 7 s, *p* = 0.000). No difference was reported in conversion, warm ischemia time, success rate, EBL, LOS and renal function up to 12 weeks post operatively between the 2 groups.

Hussein et al. [33] in their early outcomes only reported a very small series of DV (*n* = 4) versus Versius (*n* = 6) of partial nephrectomy. Versius had a shorter total operative time, EBL and conversion rate. No data were reported for post-operative complications and LOS.

### Cholecystectomy

There were 2 studies which published their outcomes on cholecystectomy: 1 multi-center RCT [37] and 1 retrospective propensity score matched analysis [36] (Table 4). Wang et al. [37] conducted a single-blinded multi-center RCT comparing early surgical outcomes of cholecystectomy performed using the DV versus the MH platform. The study had strict selection criteria and only recruited patients with benign cholelithiasis, non-inflamed gallbladder, were relatively well with low ASA scores and minimal comorbidities. All surgeons within each center were skilled surgeons in robotic cholecystectomy. There were 84 patients in each group. The mean age in the MH group was younger (45.2 ± 10.8 years vs. 48.8 ± 10.7 years, *p* = 0.028). The MH group had a lower robot-docking time (12 ± 10.5 min vs. 16.4 ± 13.9 min, *p* = 0.025) with no difference in console time. Breach of gall bladder was significantly higher in the DV group (15.7% vs. 4.8%, *p* = 0.021). No difference was noted in success rate, LOS, and post-operative complication rate.

**Table 4:** Summary findings in cholecystectomy

Study ID	EBL (ml)		Operative time (min)		LOS (days)		Post operative complications (CD all grades), *n* (%)		Breach of GB, *n *(%)	
	DV	MH	DV	MH	DV	MH	DV	MH	DV	MH
Wang [37]	7.4 ± 28.5	4.4 ± 18.1	48.6 ± 24.1	49.3 ± 20.9	7 (4–23)	7 (5–22)	43/83 (52)	42/83 (51)	13/83 (15.7)	4/83 (4.8)

	Lap	Senhance	Lap	Senhance	Lap	Senhance	Lap	Senhance	Lap	Senhance
Samalavicius [36]	11.3 ± 10.5	14.6 ± 20.8	60.8 ± 16.7	88.5 ± 24.5	1.5 ± 0.6	1.5 ± 1.1	0/20	1/20 (5)	NR	NR

*EBL* Estimated blood loss; *GB* gall bladder; *CD* Clavien–Dindo; *LOS* length of stay; *DV* Da Vinci; *MH* Micro Hand S; *Lap* laparoscopic; *NR* not reported

Samalavicius et al. [36] compared early outcomes of Senhance robotic cholecystectomy against a matching number of laparoscopic cholecystectomies within the same institution (*n* = 20). The operative time in the robotic group was significantly higher (88.5 ± 24.5 min vs. 60.8 ± 16.7 min, *p* = 0.001). No difference in EBL, LOS, and post-operative complications were detected in this small cohort study.

## Discussion

This systematic review examined 12 trials comprising of 1142 patients with the aim of evaluating the clinical outcomes of newly established multi-port robotic surgical systems. All studies included in this review were published in the last 3 years arising from Asia and Europe encompassing colorectal, urology and biliary procedures. Eight studies were head-to-head comparisons of novel robotic platforms: Micro Hand S, Senhance, Hugo^™^ RAS, and KangDuo robotic systems against the da Vinci robotic platform. The outcomes between the novel robotic systems and Da Vinci robotic system were comparable. Three studies comparing the conventional laparoscopic approach with the robotic group demonstrated longer operative time [35, 36] and lower EBL [28, 34, 35] in the robotic group.

All 8 direct comparison studies between robotic platforms included in this review showed little difference in surgical outcomes in sigmoid colectomies, rectal resections, prostatectomy, pyeloplasty, partial nephrectomy and cholecystectomy. The newly developed robotic platforms had achieved a high level of technical capabilities and mechanical precision. This is especially true in procedures which rely on superior technical execution such as the TME studies showing > 70% of complete TME [30, 35] and comparable clear resection margins in radical prostatectomy [31, 39]. However, most of the studies consisted of carefully selected patient cohorts with low BMI, minimal comorbidities, and ASA scores less than 3. Therefore, the results may not be readily applicable to the general cohort of patients with high BMI or complex pathologies. These difficult surgical circumstances can be very challenging with conventional laparoscopic techniques even for experienced surgeons and may theoretically benefit from the superior ergonomics of robotic platforms.

Unsurprisingly, when compared against conventional laparoscopic approaches, the novel robotic systems (Senhance and Micro Hand S) showed longer operative time and lower blood loss volume. The longer operative time ranged from mean increase of 3 min to 30 min [30, 34–36]. Interestingly, Kulis et al. reported a longer operative time in the first 30 cases and subsequently became shorter than the robotic group in the last 31 cases likely reflective of a steeper learning curve associated with the Senhance robotic platform consistent with a significantly higher conversion rate in the robotic group which the author attributed to the early learning curve [34].

Longer operative time has been a point of contention against robotic surgery. The length of the surgical procedure is intricately related to the learning curve when adopting a new technique. Our review demonstrated a shorter operative time with the established laparoscopic and the da Vinci robotic platforms most likely due to the surgeons’ progression beyond the learning curve with standard laparoscopic tools and the da Vinci robotic platform. Hence, as the proceduralist obtains more experience with these emerging robotic platforms and progresses beyond the learning curve, operative time should decrease over time. One study suggested a 43-min reduction in operating time after 43 cases of robotic rectal surgery [40] and Shaw et al. found a reduction in mean operating time of 53 min in robotic colorectal procedures after 15 cases, despite an increase in case complexity [41]. Looking beyond the additional minutes spent in theater, proponents of robotic surgery would argue on the point of reduced days of hospital stay. For example, Wang et al. [30] reported a mean reduction of 2.7 days in robotic TME versus laparoscopic TME. Similarly, Tewari et al.’s meta-analysis showed a mean reduction of 2.3 days in robotic versus laparoscopic prostatectomy [42].

While this review was aimed to review literature over the last 10 years, all included articles were published within the last 3 years. This indicated that all the novel robotic systems were in a similar phase of clinical development likely secondary to the lapse of several key patents in 2019 owned by Intuitive Surgical Inc. which allowed other manufacturers to introduce their new robotic systems [43]. Each new systems has been designed with different notable features (Tables 5, 6) to overcome the technical or cost constraints of the robotic platform. Furthermore, these are first generation and therefore further improvement, and optimization is expected. On the other hand, da Vinci had introduced 4 generations of their robotic system (2000/S/Si/Xi) with robust clinical data especially in the field of urology [44].

**Table 5:** Comparison of different robotic systems

	Da Vinci (Si)	Micro Hand S	Senhance	Revo-i MSR-5000	KangDuo (SR-01)	Versius	Hugo^™^
Manufacturer	Intuitive surgical	Wego	Asensus surgical	Meerecompany	Suzhou KangDuo robot	CMR surgical	Medtronic
Optics	12 mm, 3D	10 mm, 3D, HD	10 mm, 3D, HD	10 mm, 3D HD	10 mm, 3D, HD	10 mm, 3D, HD	10 mm, 3D, HD
Console/workstation	Closed	Open	Open	Closed	Open	Open	Open
Surgeon control	Finger grip, foot pedals	Finger grip, foot pedals	Laparoscopic style handles, footswitch activation	Finger grip, foot pedals	Finger grip, foot pedals	Joystick hand controls	Pistol-like handle, foot pedals
Patient console	4-armed operation cart	3-armed operation cart	4 separate modular arms	4-armed operation cart	3-armed operation cart	3–7 separate modular arms	3–4 separate modular arms
Effector arm diameter	8.4 mm	10 mm	3–10 mm	7.4 mm	10 mm	5 mm	8 mm
Notable features	Endowrist technology	Virtual haptic feedback	Eye-tracking camera, haptic feedback, instrument compatible with standard laparoscopic trocars, no direct docking required	Collision warning messages	Remote surgery via 5G (wired connection)	Haptic feedback, instrument compatible with standard laparoscopic trocars, small footprint, ergonomic console (sitting or standing position), ability to operate in 2 fields	Head tracking, haptic feedback, tilt function on effector arm
Effector arm service life	10 uses	Undisclosed	Reusable	20 uses	10 uses	13 uses	15 uses
Cost of device*	USD 1.5–2 million	Undisclosed	USD 1.3 million	Undisclosed	Undisclosed	USD 1.8-2million	USD 2.5 million

^*^Cost estimate based on public information or direct manufacturer quote. Excludes ancillary and ongoing maintenance fees

**Table 6:**
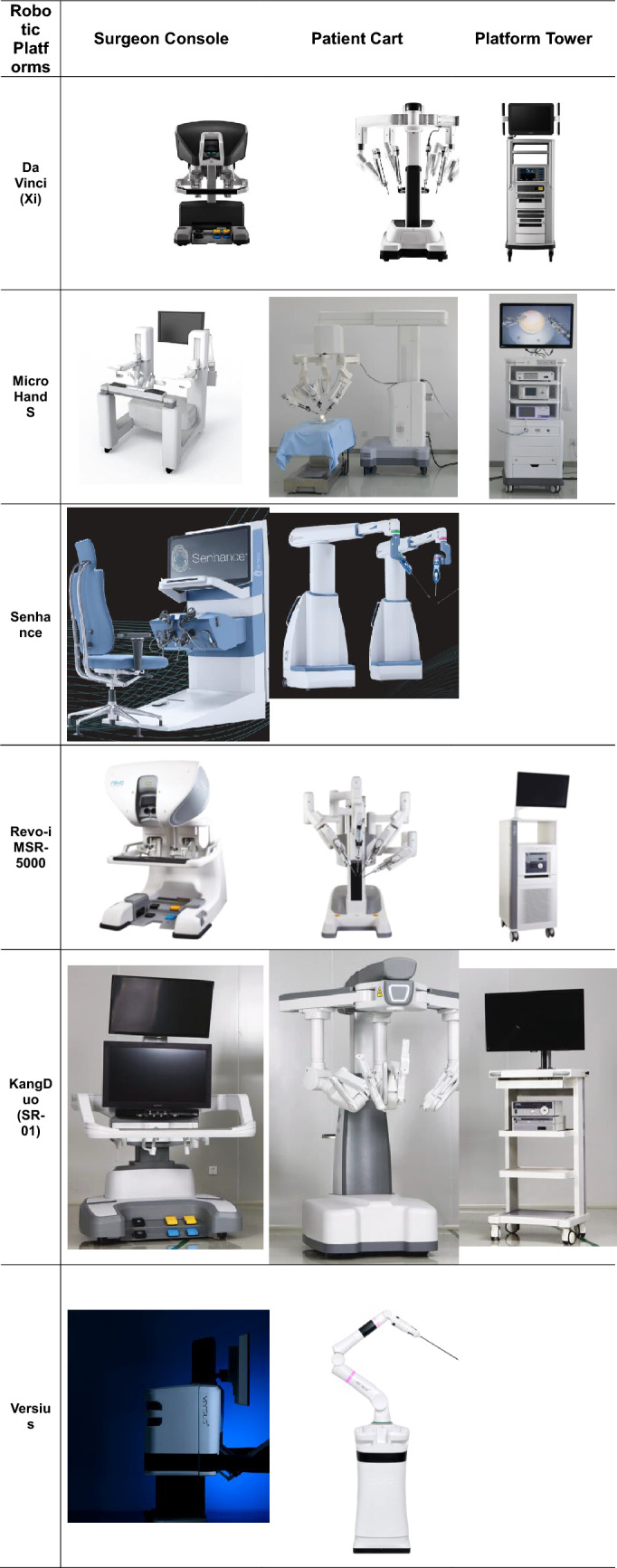
Illustrations of the various robotic platforms

Currently, the cost of robotic surgery remains a major barrier to widespread implementation [45–47], particularly in low and middle income countries even though the disease burden in these countries is significantly higher [48]. As a result, many countries such as China, Japan and Korea have developed their own robotic systems with the aim of improving cost-effectiveness. Only one study compared the hospital cost of the MH system to the DV system in sigmoid colectomy which accounted to 23.6% savings on the MH system. Other studies such as Alip et al. [31] predicted a 42% reduction of cost using the Revo-i robotic system for radical prostatectomy and Wang et al. [37] predicted a 75% reduction in cost using MH system for simple cholecystectomy. The achieved savings without compromising clinical outcomes are promising but must be interpreted with caution as these figures were not validated externally.

In addition to cost, another barrier to clinical implementation is robotic training. The learning curve for each system will be variable. Most of the procedures in this review were performed by the same group of surgeons on 2 different platforms suggesting the feasibility of skill transfer between robotic platforms. This concept has yet to be proven in clinical studies. A successful crossover of skills across different robotic platforms would open the possibility of health systems acquiring different robotic platforms to suit specific clinical circumstances without the need to retrain their robotic surgeons.

In this study, we focused our systematic review on novel multi-port soft tissue robotic systems with published comparison data to demonstrate the safe implementation and efficacy of these systems. Other emerging multi-port robotic systems with early clinical data did not fit our review criteria but possessed great potential due to their distinct design elements. SSI Mantra^™^ (Sudhir Srivastava Innovations Pvt. Ltd, Haryana, India) design featured a modular system with cardiac surgery specific instruments. Switzerland designed and manufactured Dexter robotic system (Distalmotion, Epalinges, Switzerland) used a modular platform and instruments compatible with standard laparoscopic ports. This facilitates seamless transition between laparoscopic and robotic approaches and extended the application of robotic surgery into the ‘hybrid realm’. Hinotori^™^ surgical robot system (Medicaroid Inc., Kobe, Japan) was designed with a compact operation arm that couples eight axes of motion to reduce interference between the robotic arms and bedside surgeon. Avatera robotic system (Avateramedical GmbH, Jena, Germany) featured a thin space-saving patient cart, equipped with disposable 5 mm robotic instruments. This eliminated the need for costly sterilization.

Other robotic systems such as the da Vinci’s single port robotic system which utilized a single surgical entry site and Endoquest robotic system (Endoquest Robotics, Houston, Texas, US) to conduct endoluminal procedures (i.e., submucosal dissection, endoscopic mucosal dissection) were beyond the scope of this review.

The main limitation of this review was the lack of data from RCTs. Most of the studies were retrospective case series. The retrospective nature of the studies would have inevitably introduced selection bias in our analysis with surgeons selecting surgical approaches most suitable for their skillset and robotic platform. The 2 RCTs compared patients with low ASA scores, low comorbidity state, and low BMI which limited the application in the obese and elderly population with higher burden of diseases.

## Conclusion

This systematic review highlighted the safe implementation and efficacy of 6 new robotic systems. The clinical outcomes achieved by these new robotic systems were comparable to the established da Vinci robotic system in selected cases. There is emerging evidence that these new robotic systems are reliable and present an alternative to the current available robotic platforms.

## Data Availability

The data sets used and/or analyzed in this study are available from the corresponding author upon reasonable request.

## References

[1] Hill A, McCormick J (2020) In experienced hands, does the robotic platform impact operative efficiency? Comparison of the da Vinci Si versus Xi robot in colorectal surgery. J Robot Surg 14(5):789–792 32100165 10.1007/s11701-020-01055-w

[2] Sterk MFM, Crolla RMPH, Verseveld M, Dekker JWT, van der Schelling GP, Verhoef C et al (2023) Uptake of robot-assisted colon cancer surgery in the Netherlands. Surg Endosc. 10.1007/s00464-023-10383-5 37644155 10.1007/s00464-023-10383-5 PMC10615967

[3] Bae S, Jegon W, Baek SK (2022) Single plus one-port robotic surgery using the da Vinci Single-Site Platform versus conventional multi-port laparoscopic surgery for left-sided colon cancer. Videosurgery Miniinv 17(1):179–187 10.5114/wiitm.2021.112678 PMC8886472 35251404

[4] Kaouk J, Aminsharifi A, Sawczyn G, Kim S, Wilson CA, Garisto J et al (2020) Single-port robotic urological surgery using purpose-built single-port surgical system: single-institutional experience with the first 100 cases. Urology 1(140):77–84 10.1016/j.urology.2019.11.086 32142725

[5] Samalavicius NE, Janusonis V, Siaulys R, Jasėnas M, Deduchovas O, Venckus R et al (2020) Robotic surgery using Senhance® robotic platform: single center experience with first 100 cases. J Robotic Surg 14(2):371–376 10.1007/s11701-019-01000-6 31301021

[6] Morales-Marroquin E, Khatiwada S, Xie L, de la Cruz-Muñoz N, Kukreja S, Schneider B et al (2022) Five year trends in the utilization of robotic bariatric surgery procedures, United States 2015–2019. Obes Surg 32(5):1539–1545 35169953 10.1007/s11695-022-05964-7 PMC12292007

[7] Tatarian T, Yang J, Wang J, Docimo S, Talamini M, Pryor AD et al (2021) Trends in the utilization and perioperative outcomes of primary robotic bariatric surgery from 2015 to 2018: a study of 46,764 patients from the MBSAQIP data registry. Surg Endosc 35(7):3915–3922 32737605 10.1007/s00464-020-07839-3

[8] Pastrana M, Stoltzfus J, AlMandini A, El Chaar M (2020) Evolution of outcomes of robotic bariatric surgery: first report based on MBSAQIP database. Surg Obes Relat Dis 16(7):916–922 32340825 10.1016/j.soard.2020.01.006

[9] Li ZY, Zhou YB, Li TY, Li JP, Zhou ZW, She JJ et al (2023) Robotic gastrectomy versus laparoscopic gastrectomy for gastric cancer: a multicenter cohort study of 5402 patients in China. Ann Surg 277(1):e87 34225299 10.1097/SLA.0000000000005046

[10] van Boxel GI, Ruurda JP, van Hillegersberg R (2019) Robotic-assisted gastrectomy for gastric cancer: a European perspective. Gastric Cancer 22(5):909–919 31273481 10.1007/s10120-019-00979-z PMC6694090

[11] Ojima T, Nakamura M, Hayata K, Kitadani J, Katsuda M, Takeuchi A et al (2021) Short-term outcomes of robotic gastrectomy vs laparoscopic gastrectomy for patients with gastric cancer: a randomized clinical trial. JAMA Surg 156(10):954–963 34468701 10.1001/jamasurg.2021.3182 PMC8411361

[12] Smith AJB, AlAshqar A, Chaves KF, Borahay MA (2020) Association of demographic, clinical, and hospital-related factors with use of robotic hysterectomy for benign indications: a national database study. Int J Med Robot Comput Assist Surg 16(4):e2107 10.1002/rcs.2107 PMC9206512 32276286

[13] Ghomi A, Nolan W, Sanderson DJ, Sanderson R, Schwander B, Feldstein J (2022) Robotic hysterectomy compared with laparoscopic hysterectomy: is it still more costly to perform? J Robotic Surg 16(3):537–541 10.1007/s11701-021-01273-w 34232449

[14] Doo DW, Kirkland CT, Griswold LH, McGwin G, Huh WK, Leath CA et al (2019) Comparative outcomes between robotic and abdominal radical hysterectomy for IB1 cervical cancer: results from a single high volume institution. Gynecol Oncol 153(2):242–247 30850169 10.1016/j.ygyno.2019.03.001 PMC6818647

[15] Kim DK, Park DW, Rha KH (2016) Robot-assisted partial nephrectomy with the REVO-I robot platform in porcine models. Eur Urol 69(3):541–542 26688371 10.1016/j.eururo.2015.11.024

[16] Chang KD, Abdel Raheem A, Choi YD, Chung BH, Rha KH (2018) Retzius-sparing robot-assisted radical prostatectomy using the Revo-i robotic surgical system: surgical technique and results of the first human trial. BJU Int 122(3):441–448 29645348 10.1111/bju.14245

[17] Fanfani F, Monterossi G, Fagotti A, Rossitto C, Alletti SG, Costantini B et al (2016) The new robotic TELELAP ALF-X in gynecological surgery: single-center experience. Surg Endosc 30(1):215–221 25840895 10.1007/s00464-015-4187-9

[18] Bozzini G, Gidaro S, Taverna G (2016) Robot-assisted laparoscopic partial nephrectomy with the ALF–X robot on pig models. Eur Urol 69(2):376–377 26361168 10.1016/j.eururo.2015.08.031

[19] Puntambekar SP, Rajesh KN, Goel A, Hivre M, Bharambe S, Chitale M et al (2022) Colorectal cancer surgery: by Cambridge Medical Robotics Versius Surgical Robot System—a single-institution study our experience. J Robot Surg 16(3):587–596 34282555 10.1007/s11701-021-01282-9

[20] Yao Y, Liu Y, Li Z, Yi B, Wang G, Zhu S (2020) Chinese surgical robot micro hand S: a consecutive case series in general surgery. Int J Surg 1(75):55–59 10.1016/j.ijsu.2020.01.013 31982634

[21] Gueli Alletti S, Chiantera V, Arcuri G, Gioè A, Oliva R, Monterossi G et al (2022) Introducing the new surgical robot HUGO^TM^ RAS: system description and docking settings for gynecological surgery. Front Oncol. 10.3389/fonc.2022.898060 35756633 10.3389/fonc.2022.898060 PMC9218341

[22] Bravi CA, Paciotti M, Sarchi L, Mottaran A, Nocera L, Farinha R et al (2022) Robot-assisted radical prostatectomy with the Novel Hugo robotic system: initial experience and optimal surgical set-up at a tertiary referral robotic center. Eur Urol 82(2):233–237 35568597 10.1016/j.eururo.2022.04.029

[23] Hinata N, Yamaguchi R, Kusuhara Y, Kanayama H, Kohjimoto Y, Hara I et al (2022) Hinotori surgical robot system, a novel robot-assisted surgical platform: preclinical and clinical evaluation. Int J Urol 29(10):1213–1220 35851692 10.1111/iju.14973

[24] Nakauchi M, Suda K, Nakamura K, Tanaka T, Shibasaki S, Inaba K et al (2022) Establishment of a new practical telesurgical platform using the hinotori™ Surgical Robot System: a preclinical study. Langenbecks Arch Surg 407(8):3783–3791 36239792 10.1007/s00423-022-02710-6 PMC9562055

[25] Page MJ, McKenzie JE, Bossuyt PM, Boutron I, Hoffmann TC, Mulrow CD, Shamseer L, Tetzlaff JM, Akl EA, Brennan SE, Chou R (2021) The PRISMA 2020 statement: an updated guideline for reporting systematic reviews. Int J Surg 1(88):105906 10.1016/j.ijsu.2021.105906 33789826

[26] Wells GA, Shea B, O’Connell D, Peterson J, Welch V, Losos M, et al. The Newcastle-Ottawa Scale (NOS) for assessing the quality if nonrandomized studies in meta-analyses. Available from: http://www.ohri.ca/programs/clinical_epidemiology/oxford.htm [cited 2024 Mar 22].

[27] Jadad AR, Moore RA, Carroll D, Jenkinson C, Reynolds DJM, Gavaghan DJ et al (1996) Assessing the quality of reports of randomized clinical trials: Is blinding necessary? Control Clin Trials 17(1):1–12 8721797 10.1016/0197-2456(95)00134-4

[28] Luo D, Liu Y, Zhu H, Li X, Gao W, Li X et al (2020) The MicroHand S robotic-assisted versus Da Vinci robotic-assisted radical resection for patients with sigmoid colon cancer: a single-center retrospective study. Surg Endosc 34(8):3368–3374 31482355 10.1007/s00464-019-07107-z

[29] Zeng Y, Wang G, Liu Y, Li Z, Yi B, Zhu S (2020) The, “Micro Hand S” robot-assisted versus conventional laparoscopic right colectomy: short-term outcomes at a single center. J Laparoendosc Adv Surg Tech 30(4):363–368 10.1089/lap.2019.0714 32013727

[30] Wang Y, Wang G, Li Z, Ling H, Yi B, Zhu S (2021) Comparison of the operative outcomes and learning curves between laparoscopic and “Micro Hand S” robot-assisted total mesorectal excision for rectal cancer: a retrospective study. BMC Gastroenterol 21(1):251 34098897 10.1186/s12876-021-01834-1 PMC8186043

[31] Alip S, Koukourikis P, Han WK, Rha KH, Na JC (2022) Comparing Revo-i and da Vinci in Retzius-Sparing robot-assisted radical prostatectomy: a preliminary propensity score analysis of outcomes. J Endourol 36(1):104–110 34375129 10.1089/end.2021.0421

[32] Fan S, Xiong S, Li Z, Yang K, Wang J, Han G et al (2022) Pyeloplasty with the kangduo surgical robot vs the da Vinci Si robotic system: preliminary results. J Endourol 36(12):1538–1544 35864812 10.1089/end.2022.0366

[33] Hussein AA, Mohsin R, Qureshi H, Leghari R, Jing Z, Ramahi YO et al (2022) Transition from da Vinci to Versius robotic surgical system: initial experience and outcomes of over 100 consecutive procedures. J Robotic Surg 17(2):419–426 10.1007/s11701-022-01422-9 35752748

[34] Kulis T, Hudolin T, Penezic L, Zekulic T, Saic H, Knezevic N et al (2022) Comparison of extraperitoneal laparoscopic and extraperitoneal Senhance radical prostatectomy. Robot Comput Surg 18(1):e2344 10.1002/rcs.2344 34662926

[35] Liu Y, Liu M, Lei Y, Zhang H, Xie J, Zhu S et al (2022) Evaluation of effect of robotic versus laparoscopic surgical technology on genitourinary function after total mesorectal excision for rectal cancer. Int J Surg 104:106800 35934282 10.1016/j.ijsu.2022.106800

[36] Samalavicius NE, Kaminskas T, Zidonis Z, Janusonis V, Deduchovas O, Eismontas V et al (2022) Robotic cholecystectomy using Senhance robotic platform versus laparoscopic conventional cholecystectomy: a propensity score analysis. Acta Chir Belg 122(3):160–163 33502944 10.1080/00015458.2021.1881332

[37] Wang G, Yi B, Li Z, Zhu L, Hao L, Zeng Y et al (2022) Micro-hand robot-assisted versus Da Vinci robot-assisted cholecystectomy: a multi-centre, randomized controll trial world. J Surg 46(11):2632–2641 10.1007/s00268-022-06668-w 35857076

[38] Li X, Xu W, Fan S, Xiong S, Dong J, Wang J et al (2023) Robot-assisted partial nephrectomy with the newly developed KangDuo surgical robot versus the da Vinci Si surgical system: a double-center prospective randomized controlled noninferiority trial. Eur Urol Focus 9(1):133–140 36446724 10.1016/j.euf.2022.07.008

[39] Ragavan N, Bharathkumar S, Chirravur P, Sankaran S (2023) Robot-assisted laparoscopic radical prostatectomy utilizing hugo RAS platform: initial experience. J Endourol 37(2):147–150 36205571 10.1089/end.2022.0461

[40] Byrn JC, Hrabe JE, Charlton ME (2014) An initial experience with 85 consecutive robotic-assisted rectal dissections: improved operating times and lower costs with experience. Surg Endosc 28(11):3101–3107 24928229 10.1007/s00464-014-3591-x PMC4294427

[41] Shaw DD, Wright M, Taylor L, Bertelson NL, Shashidharan M, Menon P et al (2018) Robotic colorectal surgery learning curve and case complexity. J Laparoendosc Adv Surg Tech 28(10):1163–1168 10.1089/lap.2016.0411 29733247

[42] Tewari A, Sooriakumaran P, Bloch DA, Seshadri-Kreaden U, Hebert AE, Wiklund P (2012) Positive surgical margin and perioperative complication rates of primary surgical treatments for prostate cancer: a systematic review and meta-analysis comparing retropubic, laparoscopic, and robotic prostatectomy. Eur Urol 62(1):1–15 22405509 10.1016/j.eururo.2012.02.029

[43] Rassweiler JJ, Autorino R, Klein J, Mottrie A, Goezen AS, Stolzenburg JU et al (2017) Future of robotic surgery in urology. BJU Int 120(6):822–841 28319324 10.1111/bju.13851

[44] Koukourikis P, Rha KH (2021) Robotic surgical systems in urology: what is currently available? Investig Clin Urol 62(1):14 33381927 10.4111/icu.20200387 PMC7801159

[45] Silva-Velazco J, Dietz DW, Stocchi L, Costedio M, Gorgun E, Kalady MF et al (2017) Considering value in rectal cancer surgery: an analysis of costs and outcomes based on the open, laparoscopic, and robotic approach for proctectomy. Ann Surg 265(5):960–968 27232247 10.1097/SLA.0000000000001815

[46] Park EJ, Cho MS, Baek SJ, Hur H, Min BS, Baik SH et al (2015) Long-term oncologic outcomes of robotic low anterior resection for rectal cancer: a comparative study with laparoscopic surgery. Ann Surg 261(1):129–137 24662411 10.1097/SLA.0000000000000613

[47] Di Franco G, Lorenzoni V, Palmeri M, Furbetta N, Guadagni S, Gianardi D et al (2022) Robot-assisted pancreatoduodenectomy with the da Vinci Xi: can the costs of advanced technology be offset by clinical advantages? A case-matched cost analysis versus open approach. Surg Endosc 36(6):4417–4428 34708294 10.1007/s00464-021-08793-4

[48] Olufadewa I, Adesina M, Ayorinde T (2021) Global health in low-income and middle-income countries: a framework for action. Lancet Glob Health 9(7):e899-900 34143987 10.1016/S2214-109X(21)00143-1 PMC9237759

